# Antiviral Activity of Polyene Macrolides Against Newcastle Disease Virus: Computational and Experimental Insights

**DOI:** 10.3390/molecules31111915

**Published:** 2026-06-02

**Authors:** Aidar Mukhametkaliyev, Andrey Bogoyavlenskiy, Pavel Alexyuk, Madina Alexyuk, Nadezhda Sokolova, Yergali Moldakhanov, Kuralay Akanova, Aziza Temirbayeva, Assilbek Mussoyev, Krzysztof Śmietanka, Vladimir Berezin

**Affiliations:** 1 Faculty of Veterinary and Zooengineering, Kazakh National Agrarian Research University, Almaty 050010, Kazakhstan; mukhametkalievaidar@gmail.com (A.M.);; 2 Laboratory of Antiviral Protection, Research and Production Center for Microbiology and Virology, Almaty 050010, Kazakhstan; 3 Department of Virology and Viral Animal Diseases, National Veterinary Research Institute, Al. Partyzantów 57, 24-100 Puławy, Poland

**Keywords:** Newcastle disease virus, natamycin, nystatin, filipin complex, fusion protein F, hemolysis, in silico, antiviral activity

## Abstract

The search for novel antiviral agents against Newcastle disease virus (NDV) remains a priority in industrial poultry farming due to the virus’s high contagiousness and associated economic losses, prompting evaluation of polyene macrolides as potential therapeutic candidates. We employed a comprehensive approach combining computational modeling (molecular docking and dynamics simulation) and laboratory experiments to investigate the antiviral potential of natamycin, nystatin, and filipin complex against three NDV strains. Molecular docking analysis indicated binding sites for macrolides within the hydrophobic regions of surface glycoproteins HN and F, with binding energies ranging from −6.5 to −10.5 kcal/mol, while 50 ns molecular dynamics simulation confirmed complex stability. Laboratory testing using fluorescence-based neuraminidase assays demonstrated dose-dependent inhibitory activity with IC_50_ values of 0.0043 ± 0.0015 mg/mL for filipin complex, 0.0117 ± 0.0029 mg/mL for nystatin, and 0.0220 ± 0.0138 mg/mL for natamycin, with similar ranking observed for fusion inhibition (EC_50_ values of 0.00053 ± 0.00039, 0.00545 ± 0.00560, and 0.01196 ± 0.00965 mg/mL, respectively). While filipin complex exhibited the highest antiviral activity, its significant cytotoxicity limits therapeutic application, whereas natamycin demonstrated a favorable safety profile consistent with its GRAS status. These findings indicate that natamycin exhibits a favorable safety-to-efficacy profile in vitro, warranting further in vivo investigation to clarify its mechanism of action and establish practical application protocols for NDV control in poultry.

## 1. Introduction

Actinomycetes constitute a diverse bacterial group that forms branching mycelia under favorable conditions and can exist in both aerobic and anaerobic environments [1,2]. They are widely distributed in soil, comprising over 30% of the bacterial microflora (depending on soil type and condition), and are readily isolated through plating diluted soil suspensions on standard culture media [3,4]. Members of the genus *Streptomyces* are ubiquitously distributed across diverse ecological niches, including terrestrial and aquatic ecosystems, marine sediments, and mangrove biotopes, where their metabolic activity generates the characteristic “earthy” soil aroma through geosmin production [5,6].

As one of the largest and most diverse bacterial taxa, actinomycetes synthesize a broad spectrum of bioactive metabolites, including antibiotics, antiviral compounds [7], antiparasitic agents [8], antifungal substances [9], and molecules with antitumor effects and cholesterol-lowering properties [10]. Some metabolites demonstrate neuroprotective effects in cell culture and animal model experiments [11]. Additionally, the potential role of specific actinomycete metabolites in modulating neutrophil functions is currently under active investigation [12].

Approximately two-thirds of known antibiotics and a substantial portion of antifungal medications are synthesized by actinomycete representatives, particularly *Streptomyces*, including streptomycin, tetracycline, erythromycin, chloramphenicol, neomycin, kanamycin, fosfomycin, daptomycin, clindamycin, vancomycin, nikkomycins, rapamycin, nystatin, and amphotericin B [1,11,13]. They serve as sources of numerous industrial enzymes, including proteases, amylases, peroxidases, phytases, laccases, cellulases, lipases, pectinases, glucose oxidases, lipoxygenases, and xylanases [14,15,16,17,18,19].

The endophytic actinomycete *Streptomyces* sp. LRE541 produces metabolites with antitumor activity, including neoechinulin A and two anthraquinones that induce apoptosis and cell cycle arrest in cancer cells [20].

Beyond antibacterial, antifungal, and anticancer properties, *Actinomycetota* strains also possess antiviral capabilities. These microorganisms produce compounds capable of inhibiting viral replication, opening promising avenues for developing novel antiviral treatments [21]. Since the 20th century, researchers have discovered antiviral compounds from various genera of this phylum, such as bleomycin, an antibiotic isolated by Maeda and colleagues from *Streptomyces verticillus* [22], and rifampicin, a semi-synthetic derivative of rifamycins originally isolated from *Amycolatopsis rifamycinica* [23].

One important group of antiviral compounds comprises polyene macrolides, which are cyclic amphiphilic organic molecules [24]. The polyene macrolide molecular framework consists of a large macrocyclic lactone ring (typically 20–40 carbon atoms). The amphiphilic nature of these compounds is determined by two segments: a hydrophobic segment containing a conjugated polyene chain (three to eight double bonds), and a hydrophilic segment enriched with hydroxyl groups [25,26,27]. These structural features, including macrocycle size, polyene chain length, and glycosylation presence, determine overall conformation and solubility while forming the basis of their mechanism of action.

Studies demonstrate the inhibitory effect of polyene macrolides (e.g., amphotericin B, nystatin) on the reproduction of various enveloped viruses [28,29], including vesicular stomatitis virus (VSV) [30], Japanese encephalomyelitis virus [31,32], enteroviruses [33], and SARS-CoV-2 [34,35]. Moreover, nystatin A, a tetraene-diene polyene, can inhibit HIV-1 replication in H9 cells [36]. Modern analogs like BSG005, designed for systemic application, show high activity against *Aspergillus* and *Candida* [37], opening prospects for new polyene drugs with anti-infectious and potentially antiviral activity.

Developing antiviral agents for avian pathogens remains challenging due to the high variability of avian influenza virus, where antigenic drift represents a fundamental evolutionary process [38,39]. The genetic and antigenic dynamics of the H5, H3, and H9 subtypes, including regular emergence of new lineages and variants, leads to reduced effectiveness of existing vaccines and necessitates their constant updating [40,41]. Against this background, Newcastle disease virus continues to maintain stable epizootic significance: despite widespread use of live and inactivated vaccines, disease outbreaks are regularly recorded in various regions of the world, including cases in vaccinated populations [42,43]. The genetic diversity of circulating NDV genotypes and their antigenic divergence from vaccine strains represent key reasons for periodic outbreak recurrence, highlighting the need for more reliable approaches to infection prevention and control [44,45].

## 2. Results

### 2.1. In Silico Analysis

#### 2.1.1. Molecular Docking and Initial Binding Assessment

Molecular docking calculations were performed against five Newcastle disease virus protein structures: 3T1E, 1E8V, and 3MAW (hemagglutinin HN proteins), 1G5G (F protein), and 4G1G (matrix protein).

The results of molecular docking showed that polyene macrolides possess binding energies ranging from −6.5 to −10.5 kcal/mol (Table 1). Natamycin demonstrated binding energies from −6.8 to −10.5 kcal/mol across all protein targets. Nystatin showed binding energies between −6.6 and −9.7 kcal/mol. Filipin exhibited binding affinities from −6.5 to −9.2 kcal/mol. Ribavirin demonstrated binding energies from −5.2 to −8.1 kcal/mol. Ribavirin was included in the molecular docking protocol solely as a computational reference compound. Direct energetic comparison between polyene macrolides and ribavirin is conceptually limited due to their distinct mechanisms of action and target proteins; therefore, molecular docking scores were used exclusively for internal ranking of polyene macrolides across the tested viral structures. Based on the highest binding affinity values, protein 1G5G was selected for detailed structural analysis.

#### 2.1.2. Three-Dimensional Structural Analysis (PyMOL)

**Figure 1:**
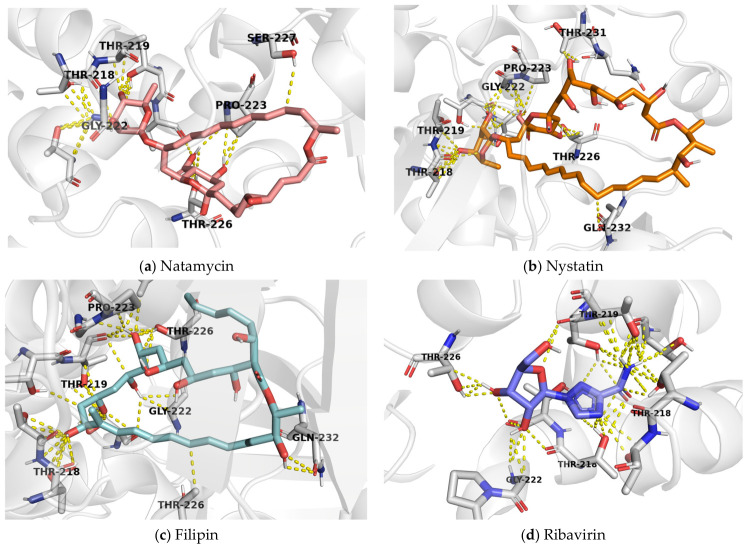
Interactions between molecules and viral protein 1G5G. (**a**) Natamycin, (**b**) nystatin, (**c**) filipin, (**d**) ribavirin. Hydrogen bonds are shown as yellow dashed lines. Protein is represented as gray ribbons with key residues labeled.

Three-dimensional visualization of polyene macrolide and ribavirin complexes with protein 1G5G, performed using PyMOL software, version 3.1.4 (Figure 1), provided detailed characterization of the spatial arrangement of ligands and features of their interaction with the protein.

Natamycin (Figure 1a) was positioned along the protein surface with its macrocyclic structure forming hydrogen bonds (shown as yellow dashed lines) with the residues THR-218, THR-219, SER-224, PRO-223, and THR-226. The compound’s extended conformation enabled simultaneous contact with multiple surface residues without penetrating internal protein cavities.

Nystatin (Figure 1b) demonstrated a similar surface-binding pattern, establishing hydrogen bond interactions with THR-218, THR-219, PRO-223, GLY-222, THR-226, and GLN-232. The polyene chain (shown in orange) was oriented along the protein surface, creating an extensive interaction network with polar and hydrophobic residues.

Filipin (Figure 1c) exhibited binding to the same general region, forming contacts with THR-218, THR-219, THR-226, GLY-222, PRO-223, and GLN-232. The compound’s structure (shown in cyan) maintained surface positioning with multiple hydrogen bond formations indicated by yellow dashed lines.

Ribavirin (Figure 1d) occupied a more localized binding area compared to the polyene macrolides. The compound (shown in blue) formed a compact interaction pattern primarily with residues THR-218, THR-219, and GLY-222, with fewer total contacts than the larger macrolide structures.

All polyene compounds demonstrated extended surface binding with multiple hydrogen bond formations, while ribavirin showed a more confined interaction pattern. The binding region represents a surface site on protein 1G5G rather than a defined active site, as functional characterization of this region has not been experimentally determined.

The identified binding region cannot be unambiguously interpreted as the protein’s active site, as experimental data on the functional role of this region are absent. In the present study, the term “binding site” is used to describe ligand localization.

#### 2.1.3. ADMET Properties and Drug-likeness Prediction

Nystatin and natamycin demonstrate elevated molecular-weight values, reflecting their large macrolide structures. The maximum ring size (MaxRing) for nystatin exceeds the values of other compounds, corresponding to the presence of complex macrocycles.

Regarding hydrogen bonding interactions, nystatin is characterized by a high number of hydrogen bond donors and acceptors (nHD, nHA), while ribavirin has relatively low values for these parameters.

Flexibility and structural rigidity (nRot, nRig) differ between the compounds: ribavirin possesses moderate flexibility and a minimal number of rigid bonds, while macrolides demonstrate more pronounced structural rigidity.

The topological polar surface area (TPSA) for nystatin is higher than for other compounds, while ribavirin is distinguished by the highest value of logarithm of solubility (logS).

The lipophilicity and distribution parameters (logP, logD_7.4_) show that filipin has the highest values, while ribavirin is characterized by minimal values for these parameters.

**Figure 2:**
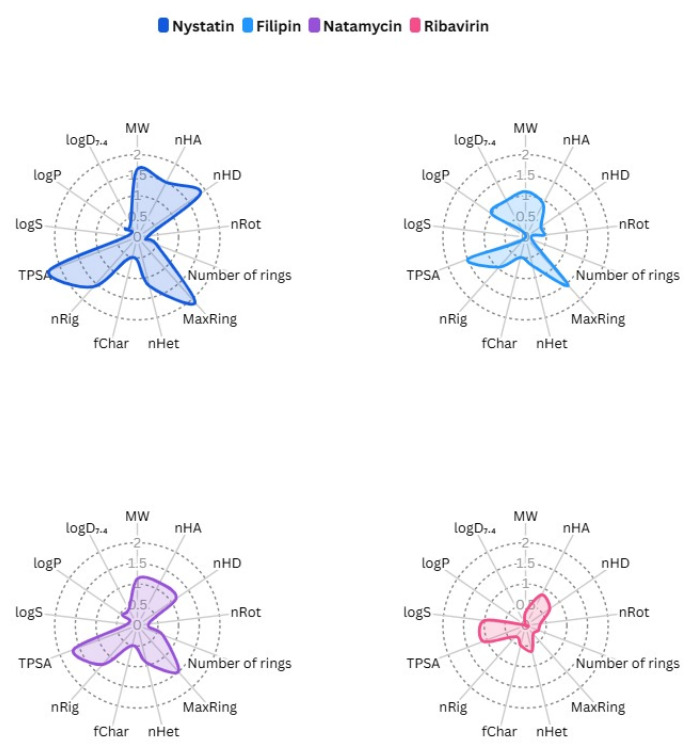
ADMET comparison of polyene macrolides (nystatin, filipin, natamycin) and ribavirin. Radar plots display 13 physicochemical properties: molecular weight (MW), hydrogen bond donors (nHD) and acceptors (nHA), rotatable bonds (nRot), rigid bonds (nRig), number of rings, maximum ring size (MaxRing), heteroatoms (nHet), formal charge (fChar), topological polar surface area (TPSA), aqueous solubility (logS), lipophilicity (logP), and distribution coefficient at physiological pH (logD_7.4_). Polyene macrolides demonstrate larger molecular size, higher rigidity, and increased polarity compared to ribavirin.

Overall, the radar chart (Figure 2) clearly demonstrates differences between polyene macrolides (nystatin, filipin, natamycin) and the low-molecular-weight compound ribavirin across a combination of structural and physicochemical properties. Polyenes are distinguished by greater molecular weight, ring size, structural rigidity, and polarity (TPSA, number of hydrogen bond donors and acceptors), as well as low aqueous solubility compared to ribavirin.

#### 2.1.4. Molecular Dynamics Simulation and System Stability Analysis

**Figure 3:**
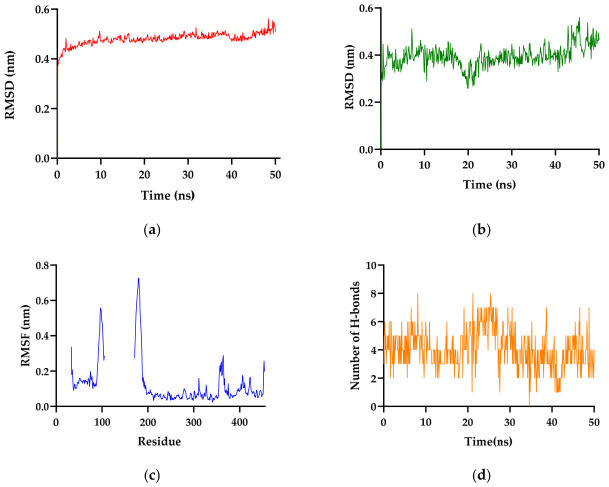
Molecular dynamics simulation analysis of the natamycin-1G5G complex during 50 ns. (**a**) The root-mean-square deviation (RMSD) of the protein backbone atoms. (**b**) The root-mean-square deviation (RMSD) of the ligand heavy atoms within the active site. (**c**) The root-mean-square fluctuations (RMSFs) of the protein amino acid residues. (**d**) Time evolution of the number of intermolecular hydrogen bonds between the ligand and the protein.

For detailed investigation of the dynamic characteristics of the 1G5G-natamycin complex and confirmation of docking data, a 50 ns molecular dynamics simulation was performed. The simulation was performed under physiological conditions (310 K, 1 atm) using explicit water solvation. Analysis of the obtained trajectory allowed evaluation of conformational stability of the system, polypeptide chain flexibility, and hydrogen bond formation patterns in the active site (Figure 3).

Root-mean-square deviation (RMSD) assessment demonstrates that the protein structure reaches equilibrium within the first 5 ns of calculation. The average deviation stabilized at 0.48 nm, which is typical for this monomeric form. Such a RMSD level is due to the presence of an extended unstructured region (residues 105–171) and high mobility of external loops. The absence of significant jumps in the curve during the final simulation segment confirms the preservation of native protein topology integrity (Figure 3a).

Natamycin’s dynamics in the binding pocket are characterized by moderate RMSD variability within 0.35–0.45 nm (Figure 3b). The stable trajectory profile demonstrates reliable ligand retention within the binding site throughout the entire observation time. A slight decrease in values observed at 20 ns reflects the mutual adaptation process between the ligand and amino acid environment. Fluctuations in the final simulation phase (40–50 ns) are local in nature and do not indicate risk of complex dissociation.

Root-mean-square fluctuation (RMSF) analysis confirmed heterogeneous flexibility of the 1G5G monomer (Figure 3c). The most labile regions correspond to structural break boundaries (residues 105 and 171), with a maximum displacement of 0.727 nm recorded for residue 179. In contrast, the central protein domain (residues 200–350) exhibits high rigidity with fluctuations less than 0.1 nm. Fragment 106–170 was excluded from analysis since it belongs to the disordered loop not resolved in the original crystallographic structure PDB ID 1G5G.

The complex stabilization mechanism was additionally studied through analysis of the intermolecular hydrogen bond networks (Figure 3d). During simulation, stable formation of three to six contacts was observed. A brief increase in bond number to eight at 8.1 ns indicates phases of highly complementary binding. Despite a single episode of contact disappearance at 34.6 ns, the system rapidly restored its initial geometry, maintaining an average of four bonds by the completion of 50 ns of simulation. Collectively, these data indicate formation of an energetically favorable and temporally stable complex suitable for sustained antiviral activity.

However, given the membrane-active nature of polyenes, the absence of an explicit membrane model in these simulations limits mechanistic inferences, as discussed in the Limitations Section.

### 2.2. In Vitro Experimental Validation

#### 2.2.1. Neuraminidase Activity Inhibition

**Figure 4:**
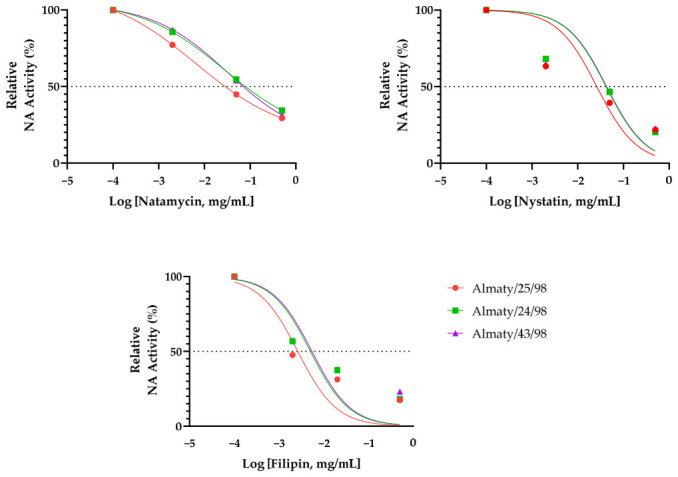
Dose–response curves for relative neuraminidase activity inhibition of NDV strains by polyene macrolides. Neuraminidase activity was measured using an ab138888 Neuraminidase Assay Kit (Fluorometric-Blue) based on enzymatic hydrolysis of NeuroBlue substrate with fluorescence detection at Ex/Em = 320/450 nm.

Analysis of the inhibitory potential of selected polyene macrolides against neuraminidase (NA) activity of Newcastle disease virus (NDV) strains revealed a concentration-dependent efficacy and structure-dependent effects of the compounds (Figure 4). The constructed nonlinear regression models indicate a dose-dependent character of enzymatic activity reduction in all investigated groups.

Natamycin demonstrated the most gradual inhibition curve slopes across the entire concentration range, corresponding to the highest IC_50_ values among tested compounds. Nystatin exhibited intermediate inhibition dynamics with moderate IC_50_ values. The filipin complex showed the most pronounced shift in the curves towards low concentrations, corresponding to the lowest IC_50_ values observed.

Quantitative analysis (Table 2) revealed the following activity ranking: filipin > nystatin > natamycin. Filipin demonstrated the highest inhibitory activity (mean IC_50_ = 0.0043 ± 0.0015 mg/mL), followed by nystatin (mean IC_50_ = 0.0117 ± 0.0029 mg/mL) and natamycin (mean IC_50_ = 0.0220 ± 0.0138 mg/mL).

Strain-specific sensitivity differences were observed, with strain Almaty/25/98 showing the highest susceptibility to all tested compounds, while strains Almaty/24/98 and Almaty/43/98 demonstrated higher resistance. High determination coefficients (R^2^ ≈ 1.0) indicate mathematical adequacy of the selected nonlinear regression models.

It should be noted that IC_50_ values represent functional potency under fixed assay conditions and do not permit the determination of kinetic parameters (Km, Vmax) or inhibition mechanisms; detailed kinetic characterization will be required to distinguish specific enzymatic inhibition from indirect effects.

#### 2.2.2. Fusion Activity Inhibition Analysis

**Figure 5:**
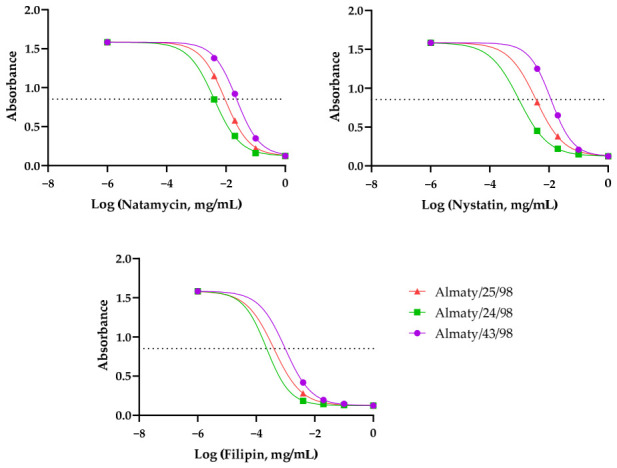
Dose–response curves for 50% effective dose inhibition (EC_50_) of fusion activity of NDV strains by polyene macrolides.

Experimental evaluation of polyene macrolide effects on viral and cellular membrane fusion revealed a distinct dependence of inhibitory effect on the structure of the investigated molecule. Despite their common chemical nature, the compounds demonstrated different efficacies in suppressing fusion activity of Newcastle disease virus strains, clearly reflected in the dose–effect curves (Figure 5) and calculated EC_50_ parameters (Table 3).

Natamycin showed the lowest activity in this experimental series. Its mean EC_50_ value was 0.01196 ± 0.00965 mg/mL. Analysis of the raw data reveals that natamycin requires significantly higher concentrations to achieve meaningful effects, with activity varying strongly between strains, confirmed by the high standard deviation.

Nystatin demonstrated more stable and potent fusion inhibition. The mean EC_50_ value was recorded at 0.00545 ± 0.00560 mg/mL, representing twice the efficacy of natamycin. Against the most sensitive isolate Almaty/24/98, nystatin showed practically nanogram-level activity values. The curve characteristics indicate that nystatin more effectively inactivates viral F-protein function at moderate therapeutic doses.

Filipin showed the most impressive results, emerging as the undisputed leader in viral fusion suppression. Its mean effective dose EC_50_ was only 0.00053 ± 0.00039 mg/mL, representing concentrations 10-fold lower than nystatin and more than 20-fold lower than natamycin for fusion blockade. Hill coefficients close to unity for all compounds additionally confirm specific blockade mechanisms rather than protein denaturation.

## 3. Discussion

This study presents a systematic evaluation of the antiviral potential of polyene macrolides against Newcastle disease virus (NDV), demonstrating dose-dependent suppression of both neuraminidase-associated activity and viral membrane fusion. However, given the well-documented membrane-active properties of polyenes [46,47], the observed effects cannot be definitively attributed to specific protein inhibition alone. While the concurrent reduction in these two functional readouts suggests a broad antiviral profile, our data do not distinguish between direct inhibition of viral glycoproteins (HN and F) and indirect effects mediated by membrane perturbation, and the contribution of membrane disruption to the observed activity remains a critical limitation of this study.

The observed experimental potency ranking (filipin > nystatin > natamycin) aligns with established structure–activity relationships for polyene macrolides, yet it presents a notable inverse relationship with molecular docking predictions. Computational analysis ranked the compounds by maximum predicted binding affinity as natamycin (−10.5 kcal/mol) > nystatin (−9.7 kcal/mol) > filipin (−9.2 kcal/mol). This discrepancy is mechanistically informative: it indicates that static protein–ligand binding energies, while useful for identifying feasible interaction sites, do not directly predict functional antiviral efficacy for membrane-active macrolides. Instead, the experimental potency gradient closely mirrors the established sterol-binding and membrane-perturbation profiles of these compounds. Filipin is known to strongly interact with membrane sterols, particularly cholesterol, resulting in pronounced membrane permeabilization [47,48], whereas natamycin exhibits more superficial sterol interactions without pore formation [49]. Consequently, the functional suppression of NDV likely arises from membrane-associated mechanisms rather than high-affinity, site-specific enzymatic inhibition. While this membrane-mediated mode of action contrasts with classical neuraminidase inhibitors such as oseltamivir, which exhibit high target specificity and minimal membrane effects, it also explains why the therapeutic applicability of highly potent polyenes may be constrained by cytotoxicity, a well-documented limitation for this compound class [50].

The high standard deviations observed in some assays (e.g., Table 2 and Table 3) reflect biological variability in viral samples and technical limitations of fluorescent-based methods. However, the dose-dependent trends (e.g., sigmoidal inhibition curves with R^2^ ≈ 1.0) remain robust, supporting the overall conclusions.

Comparison with previous antiviral studies further contextualizes the present findings. Polyene antifungals have been reported to exhibit antiviral activity against enveloped viruses, including influenza virus and herpes simplex virus. However, these studies also emphasize variability in efficacy depending on viral envelope composition and host cell context. For instance, natamycin has been reported to exhibit minimal antiviral activity in mammalian cell models despite favorable safety profiles [46], which is consistent with its lower activity ranking in the present study. Furthermore, the present study distinguishes itself by integrating computational docking and dynamics with multi-strain experimental validation within a single workflow, enabling direct evaluation of the relationship between predicted binding and observed biological activity—a linkage rarely addressed in parallel for polyene macrolides. By prioritizing natamycin, which carries GRAS status and established regulatory approval, this work also bridges mechanistic exploration with practical repurposing potential, offering a more translatable framework than prior studies focused primarily on highly cytotoxic analogs.

Molecular docking predicted stable interactions between polyene macrolides and surface glycoproteins HN and F. Ribavirin was included in the molecular docking workflow strictly as a methodological reference; however, direct comparison of binding energies is conceptually inappropriate given that ribavirin targets the viral RNA-dependent RNA polymerase rather than surface proteins [51]. Consequently, molecular docking scores should be interpreted as hypothesis-generating indicators of ligand localization rather than direct predictors of antiviral potency. This limitation aligns with broader recommendations in computational virology to avoid cross-mechanistic energetic comparisons [52].

While favorable predicted binding energies suggest polyene–protein interactions, the identified binding regions do not correspond to established sialidase active sites or characterized fusion peptide regions. This indicates that if binding contributes to antiviral activity, it likely operates through mechanisms other than direct obstruction of catalytic centers. This aligns with the known membrane-active properties of polyenes, where functional effects may arise from lipid bilayer perturbation rather than specific protein inhibition.

Molecular dynamics (MD) simulations further supported the stability of polyene–protein complexes, with sustained hydrogen bonding and limited structural deviations observed over 50 ns trajectories. The choice of a 50 ns simulation timescale is consistent with common practice in early-stage drug discovery studies, where simulations in the range of 20–100 ns are widely used to assess the initial stability of protein–ligand complexes [53,54]. For relatively rigid binding sites and ligands that do not induce large conformational rearrangements, 50 ns is generally sufficient to evaluate convergence of key parameters such as root-mean-square deviation (RMSD), root-mean-square fluctuation (RMSF), and hydrogen bonding patterns. Indeed, several studies have demonstrated that stable RMSD plateaus and persistent interaction networks within this timescale are indicative of energetically favorable binding modes [55].

The 50 ns simulations in aqueous solution were specifically designed to assess the stability of protein–ligand complexes and confirm initial binding geometry, which was successfully achieved (Figure 3). However, it is important to recognize that longer simulations (e.g., >100–500 ns or microsecond-scale) may be required to capture rare events, large conformational transitions, or ligand dissociation processes. This is particularly relevant for membrane-active compounds such as polyenes, whose primary interactions may involve lipid bilayers rather than isolated protein targets. As emphasized by recent reviews [56], simulation length should be matched to the biological question: short-to-moderate simulations are appropriate for stability assessment, whereas mechanistic elucidation often requires extended timescales and more complex system setups, including explicit membrane environments.

From a translational perspective, the repurposing of polyene macrolides represents a pragmatic strategy that aligns with recent trends in antiviral development. Natamycin, in particular, benefits from its GRAS status and established use in food preservation and veterinary medicine, which may significantly accelerate regulatory approval. However, previous attempts to repurpose polyene antifungals have been hindered by toxicity, poor solubility, and pharmacokinetic limitations [57]. Nevertheless, while natamycin’s GRAS status suggests a favorable safety profile, our study did not include direct cytotoxicity assays or selectivity index (SI) calculations, which are essential for translational applications. Therefore, while the current findings are promising, substantial optimization will be required before practical application.

Concerning the reported redox-modulating properties of polyene macrolides, nystatin has been shown to quench DPPH free radicals in EPR and UV-Vis spectroscopic assays, with UV-irradiation reducing this radical-scavenging capacity [58]. In vivo, dietary natamycin (10 mg/kg) increased serum total antioxidant capacity and glutathione peroxidase activity while reducing malondialdehyde levels in broiler chickens [59]. Additionally, polyene macrolides dose-dependently inhibited NADPH oxidase-dependent superoxide production in a cell-free enzymatic system comprising membrane and cytosolic fractions from bovine polymorphonuclear leukocytes [60]. These effects were not evaluated in the present study; given the membrane-active nature of polyenes, any contribution to anti-NDV activity is likely secondary to sterol-mediated mechanisms.

Several limitations of this study should be acknowledged. The use of purified enzymatic systems does not capture the complexity of viral infection in vivo, including host cell responses and membrane composition effects. Additionally, the study lacked critical controls: direct measurement of polyene interference with the fluorometric system (Ex/Em 320/450 nm), assessment of viral particle integrity via electron microscopy or dynamic light scattering, and positive controls with specific neuraminidase inhibitors (e.g., oseltamivir). These omissions constrain mechanistic interpretations but do not undermine the observed dose-dependent antiviral suppression. The absence of cytotoxicity and selectivity index data further limits translational applications, particularly given the membrane-disrupting properties of polyenes. Moreover, the lack of direct comparison with established NDV inhibitors restricts contextualization of these compounds in the antiviral landscape. Finally, resistance development remains to be experimentally evaluated.

While the dose-dependent inhibition of neuraminidase activity by polyene macrolides suggests functional suppression, the absence of kinetic characterization (e.g., determination of inhibition type or Kᵢ values) limits mechanistic interpretations. The observed effects may reflect direct enzyme inhibition, membrane-mediated disruption, or other indirect interactions, and further studies are required to distinguish between these possibilities.

Future work should focus on validating these findings in primary avian cell cultures and in vivo poultry models, as well as incorporating membrane-based simulation systems to better capture the mechanism of action of polyenes. Combination strategies with more specific antivirals may also enhance efficacy while reducing toxicity.

## 4. Materials and Methods

### 4.1. Viruses, Animals and Compounds

#### 4.1.1. Viruses

Paramyxoviruses: Newcastle disease virus (NDV) strains APMV-1/chicken/Almaty/24/98, APMV-1/chicken/Almaty/25/98, and APMV-1/chicken/Almaty/43/98 were provided by the virus collection of the Research and Production Center for Microbiology and Virology (Almaty, Kazakhstan). These strains are referred to as Almaty/24/98, Almaty/25/98, and Almaty/43/98, respectively, throughout this study.

Virus stocks were titrated either by cytopathic effect (CPE) on susceptible cells or by hemagglutination assays of allantois fluid.

#### 4.1.2. Animals

Ten-day-old fertilized chicken embryos (FCEs) were purchased from a licensed supplier and incubated at 37 °C under humidified conditions. All procedures involving animals were conducted in accordance with the regulations of the Ministry of Education and Science of the Republic of Kazakhstan and approved by the Committee on Ethics of Animal Experiments at the Research and Production Center for Microbiology and Virology, Almaty (protocol 02-09-184, 30 October 2023).

#### 4.1.3. Compounds

The natamycin (pimaricin, CAS: 7681-93-8), nystatin (CAS: 1400-61-9), and filipin complexes (CAS: 480-49-9) were purchased from Sigma-Aldrich (St. Louis, MO, USA) and had a purity ≥95% certified by HPLC analysis. The chemical structures were confirmed by ^1^H NMR and mass spectrometry. Stock solutions (10 mg/mL) were prepared in anhydrous dimethyl sulfoxide (DMSO, ≥99.9%, Sigma-Aldrich) under nitrogen atmosphere and stored in amber vials at −80 °C for up to 6 months.

#### 4.1.4. Buffers and Reagents

Phosphate-buffered saline (PBS, pH 7.4 ± 0.1) contained 137 mM NaCl, 2.7 mM KCl, 10 mM Na_2_HPO_4_·7H_2_O, and 1.8 mM KH_2_PO_4_ in ultrapure water (18.2 MΩ·cm, Milli-Q, Millipore, Burlington, MA, USA). All buffers were filter-sterilized (0.22 μm) and stored at 4 °C. Fresh chicken erythrocytes were harvested from 6-to-8-week-old SPF White Leghorn chickens in EDTA-containing tubes, washed three times with sterile PBS, and prepared as 5% (*v*/*v*) suspension in PBS immediately before use.

### 4.2. Experimental Methods

#### 4.2.1. In Silico Methods

##### Molecular Docking

Docking simulations were performed to model the interaction between the polyene macrolides and viral proteins. The ligand preparation was conducted in the program PyMOL 3.1.4 (https://pymol.org accessed on 8 January 2026). Docking was carried out using the AutoDock Vina version 1.2. [61,62], as well as some external tools, such as AutoDock Tools version 1.5.6. [63] The following protein structures were retrieved from the Protein Data Bank [64] and selected based on their established functional roles in the NDV life cycle: hemagglutinin-neuraminidase (HN) proteins (PDB: 3T1E, 1E8V), representing primary mediators of host–cell attachment and viral release; fusion (F) glycoproteins (PDB: 1G5G, 3MAW), essential for viral envelope–host membrane fusion; and the matrix (M) protein (PDB: 4G1G), included to assess potential interactions with structural components underlying the viral envelope. Multiple crystallographic structures for HN and F proteins were specifically analyzed to account for conformational variability and ensure the robustness of binding predictions across distinct structural states.

Natamycin (CID 5284447), filipin (CID 6433194), nystatin (CID 6420032) and ribavirin (CID 37542) were retrieved from PubChem in SDF format. Ligands were subsequently converted to PDB format for visualization in PyMOL and prepared as PDBQT files for molecular docking in AutoDock.

##### ADMET Prediction

The pharmacokinetic parameters of all ligands were predicted using the ADMETlab 3.0 platform (https://admetlab3.scbdd.com accessed on 10 December 2025). Thirteen key descriptors were evaluated, including molecular weight (MW), sum of all OH and NH groups (nHD), sum of all O and N atoms (nHA), number of rotatable bonds (nRot), ring count (nRing), largest ring size (MaxRing), heteroatom count (nHet), formal charge (fChar), number of rigid bonds (nRig), topological polar surface area (TPSA), and lipophilicity indices (logS, logP, logD_7.4_). Drug-likeness criteria were applied according to the Drug-Like Soft and Veber rules [65,66].

To compare ADMET parameters with different scales, linear min–max normalization was applied to scale all values to a 0–2 range. For each parameter, the minimum and maximum values observed across the tested compounds (nystatin, filipin, natamycin, ribavirin) were set as the lower (0) and upper (2) bounds, respectively. Individual values were then linearly transformed to fit within this range, preserving relative differences between compounds.

For further investigation of complex dynamic behavior and confirmation of protein–ligand interaction stability, the configuration with the best scoring obtained from molecular docking was selected. The selection of a specific complex for subsequent molecular dynamics analysis was based on the minimal binding free energy value and correct positioning of the molecule within the protein active site. This step enabled the transition from a static interaction model to the investigation of temporal stability of key contacts under near-physiological conditions.

##### Molecular Dynamics Simulation

Natamycin was selected for molecular dynamics analysis based on it having the highest predicted binding affinity (Table 1) and a favorable safety profile (GRAS status), prioritizing translational relevance over raw in vitro potency.

Molecular dynamics simulation of the complex based on PDB structure 1G5G was performed using GROMACS 2024.4 with the CHARMM36m force field. System preparation and ligand parameterization via SwissParam were carried out using the CHARMM-GUI interface. The monomeric protein was placed in a cubic box with TIP3P water and 0.15 M NaCl ions, maintaining a minimum clearance of 1.0 nm from box boundaries. Following energy minimization, equilibration was performed in NVT and NPT ensembles for 100 ps each at 315 K and 1 bar using the V-rescale thermostat and Parrinello–Rahman barostat.

The production phase of 50 ns duration was executed with a 2-fs time step under periodic boundary conditions (PBCs). Hydrogen bond constraints were maintained using the LINCS algorithm. Electrostatic interactions were calculated using the PME method with a cutoff radius of 1.2 nm. Trajectory preparation, including removal of periodicity effects and system centering, was performed using the gmx trjconv utility.

System stability was assessed through RMSD analysis and residue fluctuation (RMSF) analysis. Hydrogen bond analysis was conducted based on geometric criteria with a donor–acceptor distance threshold of less than 0.35 nm. All calculations were performed using the GROMACS package with subsequent data visualization in GraphPad Prism version 10.04.0.

#### 4.2.2. In Vitro Methods

##### Assessment of Viral Neuraminidase Activity Inhibition

The inhibitory potential of the nystatin, natamycin, and filipin complexes (0.5 mg/mL and 0.02 mg/mL) against neuraminidase (NA) of NDV viruses was determined using the ab138888 Neuraminidase Assay Kit (Fluorometric-Blue, Abcam, Cambridge, UK). The method is based on enzymatic hydrolysis of the NeuroBlue substrate with formation of a fluorescent product.

The reaction was performed in black 96-well microplates. To 50 μL of viral suspension, pre-incubated with the tested compounds, 50 μL of working mixture (25 μL indicator in 5 mL assay buffer) was added. After incubation (60–120 min, 37 °C) in a light-protected environment, fluorescence intensity was measured at Ex/Em = 320/450 nm. The excitation wavelength (320 nm) corresponds to the absorption maximum of the NeuroBlue chromophore, while the emission wavelength (450 nm) captures the fluorescent product peak. This wavelength combination was selected to minimize background interference and optimize the signal-to-noise ratio for neuraminidase activity detection.

##### Assessment of Fusion Activity Inhibition

The inhibitory activity of polyene macrolides (natamycin, nystatin, filipin complex) against the fusion activity of NDV strains was evaluated using hemagglutination assay with chicken erythrocytes.

Serial dilutions of the compounds were prepared in PBS using dimethyl sulfoxide (DMSO): 0.1 mg/mL, 0.02 mg/mL, and 0.004 mg/mL. Equal volumes of compounds (25 μL) and viral suspension (25 μL) were mixed and incubated for 30 min at 37 °C. Following the addition of 50 μL of 5% chicken erythrocyte suspension, samples were incubated for 30 min at 8 °C, then 1 h at 37 °C. After adding 200 μL PBS, centrifugation was performed (3000 rpm, 5 min) and the optical density of the supernatant was measured at 380 nm using a spectrophotometer.

#### 4.2.3. Data Processing and Statistical Analysis

Dose–response curves for neuraminidase and fusion inhibition assays were constructed using nonlinear regression analysis in GraphPad Prism software (version 10.04.0, San Diego, CA, USA). The raw fluorescence or absorbance data were normalized to untreated controls (defined as 100% activity) and blank wells (0% activity). IC_50_ and EC_50_ values were determined by fitting the normalized data to a sigmoidal dose–response model with variable slope. Each experiment was performed in three independent biological replicates, and results are expressed as mean ± standard deviation (SD). The coefficient of determination (R^2^) was used to assess the goodness of fit for each regression model.

## 5. Conclusions

In this study, polyene macrolides (natamycin, nystatin, and filipin complex) demonstrated dose-dependent suppression of Newcastle disease virus (NDV) neuraminidase activity and viral membrane fusion in vitro. Although computational modeling indicated stable interactions with surface glycoproteins HN and F, the identified binding regions were not experimentally confirmed as functional active sites. The absence of kinetic characterization precludes definitive claims of specific protein inhibition. Observed effects may partially reflect nonspecific membrane interactions. Given the well-established membrane-active properties of this compound class, these effects likely arise from a combination of glycoprotein association and indirect membrane-mediated mechanisms. Among the tested compounds, natamycin exhibited the most favorable balance between antiviral activity and membrane compatibility, aligning with its established safety profile and regulatory status. While these findings support the potential repurposing of natamycin for NDV management in poultry, further validation in cell-based and in vivo systems is essential to elucidate the precise mechanism of action and confirm translational relevance. The current findings demonstrate functional suppression of NDV neuraminidase and fusion, but detailed kinetic and mechanistic studies are needed to confirm the specificity of this effect.

## Figures and Tables

**Table 1:** Binding affinity of polyenes with viral proteins.

Antibiotic	Molecular Formula	Binding Affinity (kcal/mol) Max with Proteins				
		3T1E	1E8V	3MAW	1G5G	4G1G
Natamycin	C_33_H_47_NO_13_	−8.0	−6.8	−8.0	−10.5	−8.7
Filipin	C_35_H_58_O_11_	−7.6	−6.7	−6.5	−9.2	−7.5
Nystatin	C_47_H_75_NO_17_	−7.4	−6.6	−7.1	−9.7	−8.0
Ribaverin	C_8_H_12_N_4_O_6_	−6.5	−6.2	−5.2	−8.1	−6.7

3T1E, Newcastle disease virus hemagglutinin-neuraminidase protein; 1E8V, Newcastle disease virus hemagglutinin-neuraminidase protein; 3MAW, Newcastle disease virus F protein; 1G5G, Newcastle disease virus F protein; 4G1G, Newcastle disease virus matrix protein.

**Table 2:** Half-maximal inhibition (IC_50_) parameters of neuraminidase activity of NDV (mg/mL).

Compound	Almaty/25/98	Almaty/24/98	Almaty/43/98	Mean IC_50_ ± SD
Natamycin	0.0065	0.0268	0.0328	0.0220 ± 0.0138
Filipin	0.0026	0.005	0.0054	0.0043 ± 0.0015
Nystatin	0.0085	0.0124	0.0142	0.0117 ± 0.0029

**Table 3:** Half-maximal effective dose inhibition (EC_50_) parameters of NDV (mg/mL).

Compound	Almaty/25/98	Almaty/24/98	Almaty/43/98	Mean EC_50_ ± SD
Natamycin	0.00918	0.00399	0.02269	0.01196 ± 0.00965
Filipin	0.00039	0.00022	0.00097	0.00053 ± 0.00039
Nystatin	0.00363	0.00096	0.01174	0.00545 ± 0.00561

## Data Availability

The original contributions presented in this study are included in the article. Further inquiries can be directed to the corresponding author.
